# Supplementary material for: Association between quantitative sensory testing and pain or disability in paediatric chronic pain: protocol for a systematic review and meta-analysis
Source: BMJ Open. 2019 Oct 7;9(10):e031861. doi: 10.1136/bmjopen-2019-031861 (PMC6797335; doi:10.1136/bmjopen-2019-031861)
Supplement: Author's manuscript [file bmjopen-2019-031861.draft_revisions.pdf]

BMJ Open is committed to open peer review. As part of this commitment we make the peer review history of every article we publish publicly available.

When an article is published we post the peer reviewers' comments and the authors' responses online. We also post the versions of the paper that were used during peer review. These are the versions that the peer review comments apply to.

The versions of the paper that follow are the versions that were submitted during the peer review process. They are not the versions of record or the final published versions. They should not be cited or distributed as the published version of this manuscript.

BMJ Open is an open access journal and the full, final, typeset and author-corrected version of record of the manuscript is available on our site with no access controls, subscription charges or pay-per-view fees (http://bmjopen.bmj.com).

If you have any questions on BMJ Open's open peer review process please email info.bmjopen@bmj.com

BMJ Open

The association between quantitative sensory testing and pain or disability in paediatric chronic pain: Protocol for a systematic review and meta-analysis

Journal:	*BMJ Open*
Manuscript ID	bmjopen-2019-031861
Article Type:	Protocol
Date Submitted by the Author:	22-May-2019
Complete List of Authors:	Schoth, Daniel; University of Southampton, School of Psychology<br>Blankenburg, Markus; University of Witten/Herdecke, Faculty of Health<br>Wager, Julia; Universitat Witten/Herdecke,<br>Broadbent, Philippa; University of Southampton, School of Psychology<br>Zhang, Jin; University of Southampton Faculty of Engineering and the Environment, School of Psychology<br>Zernikow, Boris; Universität Witten/Herdecke<br>Liossi, Christina; University of Southampton, School of Psychology
Keywords:	Quantitative sensory testing, QST, Paediatric chronic pain, Disability, Systematic review, Meta-analysis

SCHOLARONE™
Manuscripts

The association between quantitative sensory testing and pain or disability in paediatric chronic pain: Protocol for a systematic review and meta-analysis

Schoth D. E.[1], Blankenburg M.[2,3], Wager J.[4,5], Broadbent P[1]., Zhang J[1]., Zernikow B.[4,5], and Liossi C.[1,2]

[1]Pain Research Laboratory, School of Psychology, University of Southampton, Highfield, Southampton, UK; [2] Department for Pediatric Neurology, Child Pain Center Baden-Württemberg, Klinikum Stuttgart, Germany; [3] Witten/Herdecke University, Faculty of Health, Witten, Germany; [4] German Paediatric Pain Centre, Children's and Adolescents' Hospital Datteln, Germany; [5] Department of Children's Pain Therapy and Paediatric Palliative Care, Witten/Herdecke University, Faculty of Health, School of Medicine, Witten, Germany; [6] Great Ormond Street Hospital for Children NHS Foundation trust, London, UK.

Corresponding author: Prof. Christina Liossi, Pain Research Laboratory, Department of Psychology, University of Southampton, Highfield, Southampton, SO17 1BJ, UK. E-mail: C.Liossi@soton.ac.uk, Tel: 0044 (0)238059 4645.

For peer review only - http://bmjopen.bmj.com/site/about/guidelines.xhtml

Abstract

Introduction. This protocol describes the objective and methods of a systematic review of the association between quantitative sensory testing (QST) measures and pain intensity or disability in paediatric chronic pain (PCP). The review will also assess whether the relationship strength is moderated by variables related to the QST method and pain condition; the use of QST in PCP (modalities, outcome measures and anatomical test sites as well as differentiating between pain mechanisms (e.g., neuropathic vs. nociceptive) and in selecting analgesics); the reliability of QST across the paediatric age range; the ability of QST to differentiate patients with chronic pain from healthy controls; and differences between anatomical test sites. **Methods and analysis.** MEDLINE, PsycINFO , CINHAL, Web of Science, Scopus, Cochrane Library and OpenGrey will be searched. English language studies will be eligible if they; recruit a sample aged 6 to 24 (inclusive) with chronic pain, including primary and secondary pain; apply at least one of the following QST modalities: chemical, electrical, mechanical (sub-groups include pressure, punctate/brush, vibratory) or thermal stimulus, to measure perception of noxious or innocuous stimuli applied to skin, muscle or joint; use a testing protocol to control for stimulus properties: modality, anatomical site, intensity, duration, and sequence. Following title and abstract screening, the full texts of relevant records will be independently assessed by two reviewers. For eligible studies, one reviewer will extract study characteristics and data, and another will check for accuracy. Both will undertake independent quality assessments using Appraisal Tool for Cross Sectional Studies (AXIS). A qualitative synthesis will be presented with discussion centred around different QST modalities. Where eligible data permit, meta-analyses will be performed separately for different QST modalities using Comprehensive Meta-Analysis. **Ethics and dissemination.** Review findings will be reported in a peer-reviewed journal and presented at conferences. The study raises no ethical issues.

For peer review only - http://bmjopen.bmj.com/site/about/guidelines.xhtml

Article Summary

Strengths and Limitations of this Study

- This systematic review can give us a greater understanding of the association between quantitative sensory testing (QST) (in accordance with either the DFNS (German research network on Neuropathic Pain) protocol or other protocols) and pain intensity or disability in paediatric chronic pain and could inform the use of QST in clinical practice
- This systematic review will follow robust guidelines and the quality of included articles will be assessed using validated tools
- The heterogeneity of the included studies, which may use different quantitative sensory measures across different chronic pain conditions and age range, could limit the overall data synthesis

Keywords

Quantitative sensory testing; QST; Pediatric chronic pain; Disability; Systematic review; Meta-analysis

For peer review only

Introduction

Paediatric chronic pain (PCP) is defined as persistent or recurring pain of any aetiology lasting for longer than three months.[1 2] A cross-sectional study of 561 schoolchildren aged 8 to 16 (mean age 11.89) showed a PCP prevalence of 37.3%, of which 29.3% reported pain in two or more sites.[1] Prevalence rates do vary across reports and conditions however, as demonstrated in a systematic review of 41 articles (e.g., 4% – 40% for musculoskeletal pain, 8% – 83% for headache, and 4% – 53% for abdominal pain) which may be due, in part, to inconsistent definitions of chronic pain adopted by individual studies [3]. An analysis of the 2016 National Survey of Children's Health in the United States estimated a population prevalence of PCP to be 6% based on a sample of 43,712 children aged 0 to 17 years.[4]

Despite variations in precise prevalence estimates, PCP is a significant clinical problem worldwide[5], and can have enormous impacts on the child's quality of life.[6] Compared to their healthy peers children with chronic pain report substantially worse quality of life in physical, emotional, social, and school functioning[7], and frequently experience disordered sleep.[8] A purely biomedical model of PCP is outdated, and instead the severity, impact and experience of chronic pain is now recognised to be shaped by a complex interaction of biological, psychological and social factors.[9] Multimodal interdisciplinary interventions are therefore essential in the treatment of severe PCP.[10 11] A recent review of 21 studies highlights the effectiveness of interdisciplinary interventions in PCP management. Patients receiving interdisciplinary interventions reported significant improvements pre- to post-intervention in pain intensity, functional disability, anxiety, depression, catastrophizing, school attendance, school functioning and pain acceptance, and significantly lower pain intensity 0-1 month post-intervention compared to patients randomised to control groups.[12]

Quantitative sensory testing (QST) refers to a range of non-invasive techniques for exploring somatosensory processing, including positive and negative sensory phenomena.[13 14] QST is psychophysical in nature, relying on the individual's subjective report to a standardized stimulus.[13 15] QST might provide unique information about the functional status of the somatosensory system and

For peer review only - http://bmjopen.bmj.com/site/about/guidelines.xhtml

therefore better guide pain treatment. Although QST is commonly used in adult populations, QST is also considered feasible, reliable and reproducible in children, including children as young as four years old when using thermal stimuli.[16] In recent years a small but growing number of published studies have explored the use of QST in PCP populations, although calls have been made to expand this further.[17] Similar to other psychophysical methods, and to enable the comparison to published normative values and between different QST labs as well as follow-up examinations, QST requires the active participation of the young patient, a standardized assessment using standardized instructions and calibrated stimuli applied by highly trained investigators. A number of protocols are followed to this end with the protocol proposed by the German Research Network on Neuropathic Pain (DFNS) the most well developed. DFNS includes examination of warm and cold detection and pain thresholds including the assessment of paradoxical heat sensation during alternating temperature stimuli (using a thermostat), mechanical detection thresholds for light touch (using a set of von Frey filaments), and for vibration (using a Rydel-Seifter tuning fork), mechanical pain thresholds for pinprick (using pinprick stimulators of different intensities) and for blunt pressure (using a pressure algometer) as well as a stimulus-response function for mechanical pain sensitivity to pinprick stimuli, dynamic mechanical allodynia for the stimuli of different intensities (using a cotton wool, a Q tip and a standardized brush) and temporal summation for pinprick stimuli.[18]

The primary objective of this systematic review and meta-analysis is to examine the relationship between established QST measures and pain intensity or disability in PCP. The second objective is to assess whether the strength of the relationship is moderated by variables related to the QST method and pain condition. Furthermore, this systematic review and meta-analysis will examine: [1] the use of QST in PCP (modalities, outcome measures and anatomical test sites differentiating between pain mechanisms (e.g., neuropathic vs nociceptive) and in selecting analgesics; [2] the reliability of QST across the paediatric age range; [3] the ability of QST to differentiate patients with chronic pain from healthy controls, and [4] differences between anatomical test sites.

For peer review only - http://bmjopen.bmj.com/site/about/guidelines.xhtml

Methods

This protocol was developed in line with PRISMA-P guidelines[19 20] and is registered with PROSPERO (CRD42019134069), an international register of systematic reviews. Any amendments to the protocol will be recorded on PROSPERO.

Literature Search

We will search the following electronic bibliographic databases: MEDLINE, PsycINFO, CINHAL (title, abstract), Web of Science (title), Scopus (article title, abstract, keywords), and Cochrane Library (title, abstract, keywords). A search of non-traditional publications, commonly known as 'grey literature', will be conducted using Open Grey. All databases will be searched from database inception to the date of search. Only studies published in the English language will be included. The reference lists of all eligible papers will be hand searched for relevant studies. The search strategy will include three concept blocks pertaining to the type of pain (*pain**, *ache**, *abdominal pain*, *arthritis*, *headache**, *musculoskeletal pain*, *fibromyalgia*), study population (*boy**, *girl**, *child**, *teenager**, *youth**, *adolescen**, *young**, *schoolchild**, *school child**, *juvenil**, *paediatric*, *pediatric*), and quantitative sensory testing (*quantitative sensory test**, *sensory test**, *QST*, *threshold*, *tolerance*, *pressure*, *electrical*, *warm*, *heat*, *hot*, *cool*, *cold*, *mechanical*, *hyperalgesia*, *hyperaesthesia*, *allodynia*, *sensitisation*, *sensitivity*).

Inclusion Criteria

Studies will be eligible for inclusion if they meet the following criteria:

1) Available in the English language.
2) Recruits a sample aged 6 to 24 (inclusive) with chronic pain, including primary pain (i.e., pain in one or more anatomic regions that has lasted for longer than 3 months and cannot be better explained by any other chronic pain condition) and secondary pain (i.e., pain that has lasted longer than 3 months that is symptomatic of another health condition).[21] In cases where the sample age range includes individuals older than 24 years of age, the record will only be

For peer review only - http://bmjopen.bmj.com/site/about/guidelines.xhtml

eligible for inclusion if separate data is presented for the age group of interest to the present review. While the adolescent age range typically extends to 18 years, the transition period from childhood to adulthood is now argued to occupy a greater portion of the life course than before, and therefore an upper age limit of 24 years corresponds more closely to adolescent growth and popular understandings of this life phase.[22]

3) Applies at least one of the following QST modalities: chemical, electrical, mechanical (sub-groups will include pressure, punctate/brush, and vibratory) or thermal stimulus, in order to measure perception of noxious or innocuous stimuli applied to skin, muscle or joint.

4) Uses a testing protocol to control for stimulus properties: modality, anatomical site, intensity, duration, and sequence.

QST is defined as a method that quantifies the magnitude of physical stimuli (e.g., pressure, heat, cold, vibration, electrical current) that is required to determine a specific perception (i.e., threshold, tolerance, temporal summation, magnitude rating, wind-up, limen). For inclusion in this systematic review, the application of the physical stimulus will have to be standardized and the physical stimulus will have to be expressed in quantitative terms (e.g., pressure: kg/cm^2; heat/cold: ˚C). The evoked sensory and pain perception will have to be reported subjectively by the participant (e.g., yes/no for pin prick threshold) and quantitatively (e.g., pressure: kg/cm^2; heat/cold: ˚C; intensity ratings using VAS, NRS or other validated scales). Studies using invasive forms of QST will excluded. Correlation coefficients (Pearson's *r* or Spearman's *q*) for the relationship between QST measured locally and/or at a remote site and pain intensity/disability will have to be reported or could be calculated from the data reported in the study or from data obtained from the study authors. Studies that only provide correlations between pain intensity/disability and QST composite scores (i.e., local and remote site combined) will be excluded.

Data Extraction

The titles and abstracts of retrieved records will be transferred into the digital reference manager Endnote X8. Titles will be scanned for duplication using an automated search engine within

For peer review only - http://bmjopen.bmj.com/site/about/guidelines.xhtml

Endnote, and any duplicates will be removed. Records will then be screened by one review team member to identify studies that potentially meet the inclusion criteria, with a second review team member screening a random selection of 10% of records. The full texts of potentially relevant records will be retrieved, which will be independently assessed for eligibility by two review team members. Any disagreement between reviewers over study eligibility will be resolved through discussion with a third review author. Data will be extracted using a pre-piloted template. Extracted information will include the country in which the research was conducted; demographics and medical characteristics of PCP patients; demographics of healthy controls (where applicable); QST protocol details; the training of the QST administrators; the outcome measures recorded; and QST and other outcome results. For eligible studies, one reviewer will perform extraction of study characteristics and data, and another will check for accuracy (with a third review author where necessary). If information is missing or unclear, the corresponding author for the study in question will be contacted.

Study Quality Assessment

Two review authors will independently assess study quality using Appraisal Tool for Cross Sectional Studies (AXIS).[23] AXIS features 20 questions which are answered as either 'yes', 'no' or 'don't know', along with spaces for comments on each judgement provided. Although similar to the STROBE statement (a 22-item checking for use with observational (cohort, cross-sectional, case-control) studies[24]), AXIS provides an assessment of both study design and risk of bias. Interrater reliability between the two reviewers will be calculated using Cohen's Kappa, where a Kappa value of .80 or above is considered a strong level of agreement and indicates high interrater reliability.[25] Any disagreements between the review authors will be resolved by discussion, with involvement of a third review author where necessary.

Statistical Analysis

All statistical analyses will be performed using Comprehensive Meta-Analysis (CMA, version 3.0[26]) and Power and Sample Size Calculations 3.0.43 software. CMA converts correlation coefficients to Fisher's z scores, with all analyses performed using these transformed values.[27]

For peer review only - http://bmjopen.bmj.com/site/about/guidelines.xhtml

Similar to a recent review of QST in spinal pain[28] Pearson's *r* or Spearman's rank coefficient will be synthesized together. Unlike Pearson's product-moment correlation coefficient, which requires the assumption that the relationship between the variables is linear and variables to be measured on interval scales, Spearman's rank correlation coefficient is a nonparametric (distribution-free) rank statistic. It assesses how well an arbitrary monotonic function can describe a relationship between two variables, without making any assumptions about the frequency distribution of the variables and can be used for variables measured at the ordinal level. We will use .25, .5, and .75 as cut-off points to interpret the strength of the relationship as little or zero (.00 to .25), fair (.26 to .50), moderate to good (.51 to .75), and good to excellent (above .75).[29]

Heterogeneity will be examined using Cochrane's Q test and the I^2 statistic. With Cochrane's Q, a significant result is indicative of heterogeneity. The I^2 statistic describes the percentage of variability in effect estimates due to heterogeneity as opposed to sampling error [30]. The Cochrane Handbook provides the following rough guide for interpretation of the I^2 statistic: 0% to 40%: might not be important; 30% to 60%: may represent moderate heterogeneity; 50% to 90%: may represent substantial heterogeneity; 75% to 100%: considerable heterogeneity[30] Given sufficient availability of data, mixed effects meta-regression will be used to formally assess the effect of the potential categorical predictors (QST testing site, pain condition, pain type, type of pain induction stimulus) on the strength of the relationship between pain threshold and pain intensity/disability. The effect of each categorical predictor will be tested in a separate meta-regression.

The ability of QST to detect somatosensory differences between young people with chronic pain and healthy controls will be assessed using standardised mean differences (SMDs). The standardised mean difference will be calculated as the mean difference between groups divided by the standard deviation of the specific QST measure for all participants pooled across both groups. The standardised mean difference expresses the difference between groups in QST measures as multiples of the observed standard deviation. The standardised mean difference is a ratio, with numerator and denominator in the same units as the original measurement, therefore has no units, does not depend on

For peer review only - http://bmjopen.bmj.com/site/about/guidelines.xhtml

the original measurement scale, and allows a direct comparison of the effect across studies that used different QST measures. By convention, a SMD or $d = 0.2$ is considered to be small, $d= 0.5$ moderate and $d = 0.8$ large in size.[31]

Each study will be screened for intraclass correlation coefficients (ICCs) describing the test-retest reliability of QST. The reliability of the measurement across test occasions will be rated excellent if the ICC > .75, adequate if .40 – .74, and poor if < .40.[32] Due to developmental and sex differences in somatosensory perception[33 34] data will be grouped and analysed separately where possible for males and females, and by appropriate age groups. Data will also be grouped into local (i.e., the site of pain) and non-local (i.e., a non-painful site) categories. For more than 10 studies, we will use funnel plots where appropriate to detect potential reporting biases and small-study effects. We will also conduct sensitivity analyses where appropriate to verify the robustness of the study conclusions.

Patient and public involvement

Consultation with young people, parents and health care professionals has been used to determine their perception of the clinical utility of quantitative sensory testing in chronic pain. It is based on their perspectives that this systematic review was deemed timely and crucial to conduct to inform further research work and implementation of QST in routine clinical practice.

Discussion

This systematic review will be the first to investigate the association between QST and pain intensity or disability in paediatric chronic pain. The review findings will be used to inform our ongoing work to develop a core outcome set for specialist paediatric chronic pain services across the UK.

Ethics and dissemination

As this is a systematic review of published literature, ethical approval will not be sought. We will publish the protocol and our findings in peer-reviewed journals aimed at paediatric chronic pain

For peer review only

clinicians and researchers as well as health commissioners. We will present our work at national and international meetings focused on paediatric chronic pain.

Acknowledgements

We would like to thank Holly Brown, Navin Down, Victoria Gibbons and Olivia Ranger (BSc Psychology students studying at the University of Southampton who undertook their 3rd year dissertation projects at the Pain Research Laboratory) for performing a scoping review of relevant literature that informed the development of this protocol.

Author Contributions

CL conceived the idea, planned and designed the study protocol. CL and DS planned the data extraction and statistical analysis and wrote the first draft; MB, JW, and BZ JB and LB provided critical insights. All authors have approved and contributed to the final written manuscript.

Funding

This development of this protocol was supported by ESRC (Grant number: RES-000-22-4128). This funding source had no role in the design of this review and will not have any role during its execution, analyses, interpretation of the data, or decision to submit results.

Competing Interests

None declared.

For peer review only

References

1. Huguet A, Miró J. The severity of chronic pediatric pain: an epidemiological study. J Pain 2008;**9**(3):226-36.

2. Merskey H, & Bogduk, N. *Classification of chronic pain: Descriptions of chronic pain syndromes and definitions of pain terms*. Seattle: IASP Press 1994.

3. King S, Chambers CT, Huguet A, et al. The epidemiology of chronic pain in children and adolescents revisited: a systematic review. Pain 2011;**152**(12):2729-38 doi: 10.1016/j.pain.2011.07.016.

4. Tumin D, Drees D, Miller R, et al. Health Care Utilization and Costs Associated With Pediatric Chronic Pain. J Pain 2018;**19**(9):973-82 doi: 10.1016/j.jpain.2018.03.012.

5. Miró J, McGrath PJ, Finley GA, et al. Pediatric chronic pain programs: current and ideal practice. Pain Reports 2017;**2**(5):e613 doi: 10.1097/PR9.0000000000000613.

6. Rolfe PM. Paediatric chronic pain. Anaesthesia & Intensive Care Medicine 2016;**17**(11):531-35.

7. Varni JW, Limbers CA, Burwinkle TM. Impaired health-related quality of life in children and adolescents with chronic conditions: a comparative analysis of 10 disease clusters and 33 disease categories/severities utilizing the PedsQL 4.0 Generic Core Scales. Health Qual Life Outcomes 2007;**5**:43 doi: 10.1186/1477-7525-5-43.

8. Evans S, Djilas V, Seidman LC, et al. Sleep Quality, Affect, Pain, and Disability in Children With Chronic Pain: Is Affect a Mediator or Moderator? J Pain 2017;**18**(9):1087-95 doi: 10.1016/j.jpain.2017.04.007.

9. Liossi C, Howard RF. Pediatric chronic pain: biopsychosocial assessment and formulation. Pediatrics 2016;**138**(5):e20160331.

10. Rajapakse D, Liossi C, Howard RF. Presentation and management of chronic pain. Arch Dis Child 2014;**99**(5):474-80 doi: 10.1136/archdischild-2013-304207

11. Odell S, Logan DE. Pediatric pain management: the multidisciplinary approach. J Pain Res 2016;**6**:785–90. doi: 10.2147/JPR.S37434.

For peer review only - http://bmjopen.bmj.com/site/about/guidelines.xhtml

12. Liossi C, Johnstone L, Lilley S, et al. Interdisciplinary interventions for pediatric chronic pain: A systematic review and meta-analysis. Br J Anaesth in press.

13. Yarnitsky D. Quantitative Sensory Testing. Muscle Nerve 1997;**20**(2):198 - 204.

14. Yarnitsky D, Pud D. Quantitative sensory testing. In: Binnie CD, Cooper R, Mauguiere F, et al., eds. Clinical neurophysiology: EMG, nerve conduction and evoked potentials, vol1. Amsterdam: Elsevier, 2004:305 - 28.

15. Zaslansky R, Yarnitsky D. Clinical applications of quantitative sensory testing (QST). J Neurol Sci 1998;**153**(2):215-38 doi: https://doi.org/10.1016/S0022-510X(97)00293-1.

16. Hilz MJ, Glorius SE, Schweibold G, et al. Quantitative thermal perception testing in preschool children. Muscle & Nerve: Official Journal of the American Association of Electrodiagnostic Medicine 1996;**19**(3):381-83.

17. Tham SW, Palermo TM, Holley AL, et al. A population-based study of quantitative sensory testing in adolescents with and without chronic pain. Pain 2016;**157**(12):2807-15 doi: 10.1097/j.pain.0000000000000716.

18. Rolke R, Baron R, Maier C, et al. Quantitative sensory testing in the German Research Network on Neuropathic Pain (DFNS): Standardized protocol and reference values. Pain 2006;**123**(3):231-43.

19. Moher D, Shamseer L, Clarke M, et al. Preferred reporting items for systematic review and meta-analysis protocols (PRISMA-P) 2015 statement. Systematic Reviews 2015;**4**(1):1.

20. Shamseer L, Moher D, Clarke M, et al. Preferred reporting items for systematic review and meta-analysis protocols (PRISMA-P) 2015: elaboration and explanation. BMJ 2015;**349**:g7647 doi: 10.1136/bmj.g7647

21. Treede R-D, Rief W, Barke A, et al. A classification of chronic pain for ICD-11. Pain 2015;**156**(6):1003 doi: 0.1097/j.pain.0000000000000160.

22. Sawyer SM, Azzopardi PS, Wickremarathne D, et al. The age of adolescence. The Lancet: Child and Adolescent Health 2018;**2**(3):223 - 28 doi: 10.1016/S2352-4642(18)30022-1.

For peer review only - http://bmjopen.bmj.com/site/about/guidelines.xhtml

23. Downes MJ, Brennan ML, Williams HC, et al. Development of a critical appraisal tool to assess the quality of cross-sectional studies (AXIS). BMJ Open 2016;**6**(12):e011458 doi: 10.1136/bmjopen-2016-011458.

24. Von Elm E, Altman DG, Egger M, et al. The Strengthening the Reporting of Observational Studies in Epidemiology (STROBE) statement: guidelines for reporting observational studies. PLoS Med 2007;**4**(10):e296.

25. McHugh ML. Interrater reliability: The kappa statistic. Biochemia Medica 2012;**22**(3):276-82.

26. Comprehensive Meta-Analysis Version 3 [program]. Englewood, New Jersey: Biostat, 2013.

27. Borenstein M, Hedges LV, Higgins J, et al. *Introduction to meta-analysis*. Cornwell: Wiley Online Library, 2009.

28. Hübscher M, Moloney N, Leaver A, et al. Relationship between quantitative sensory testing and pain or disability in people with spinal pain—a systematic review and meta-analysis. PAIN® 2013;**154**(9):1497-504.

29. Portney LG. *Foundations of Clinical Research: Applications to Practice 3th (third) Edition*. New Jersey: Pearson Education, 2009.

30. Higgins JPT, Green S. *Cochrane Handbook for Systematic Reviews of Interventions*: The Cochrane Collaboration, 2008.

31. Cohen J. *Statistical power analysis for the behavioral sciences, 2nd edition* Hillsdale: Erlbaum, 1988.

32. Fleiss JL, Levin B, Paik MC. *Statistical Methods for Rates and Proportions*: John Wiley & Sons, 2013.

33. Blankenburg M, Boekens H, Hechler T, et al. Reference values for quantitative sensory testing in children and adolescents: Developmental and gender differences of somatosensory perception. Pain 2010;**149**(1):76-88 doi: http://dx.doi.org/10.1016/j.pain.2010.01.011.

34. Blankenburg M, Meyer D, Hirschfeld G, et al. Developmental and sex differences in somatosensory perception—a systematic comparison of 7- versus 14-year-olds using quantitative sensory testing. . Pain 2011;**152**(11):2625 - 31 doi: 10.1016/j.pain.2011.08.007.

For peer review only

For peer review only - http://bmjopen.bmj.com/site/about/guidelines.xhtml

PRISMA-P (Preferred Reporting Items for Systematic review and Meta-Analysis Protocols) 2015 checklist: recommended items to address in a systematic review protocol*

Section and topic	Item No	Checklist item	Page
**ADMINISTRATIVE INFORMATION**			
Title:			
Identification	1a	Identify the report as a protocol of a systematic review	1
Update	1b	If the protocol is for an update of a previous systematic review, identify as such	N/A
Registration	2	If registered, provide the name of the registry (such as PROSPERO) and registration number	6
Authors:			
Contact	3a	Provide name, institutional affiliation, e-mail address of all protocol authors; provide physical mailing address of corresponding author	1
Contributions	3b	Describe contributions of protocol authors and identify the guarantor of the review	11
Amendments	4	If the protocol represents an amendment of a previously completed or published protocol, identify as such and list changes; otherwise, state plan for documenting important protocol amendments	N/A
Support:			
Sources	5a	Indicate sources of financial or other support for the review	11
Sponsor	5b	Provide name for the review funder and/or sponsor	11
Role of sponsor or funder	5c	Describe roles of funder(s), sponsor(s), and/or institution(s), if any, in developing the protocol	11
**INTRODUCTION**			
Rationale	6	Describe the rationale for the review in the context of what is already known	4-5
Objectives	7	Provide an explicit statement of the question(s) the review will address with reference to participants, interventions, comparators, and outcomes (PICO)	5
**METHODS**			
Eligibility criteria	8	Specify the study characteristics (such as PICO, study design, setting, time frame) and report characteristics (such as years considered, language, publication status) to be used as criteria for eligibility for the review	6-7
Information sources	9	Describe all intended information sources (such as electronic databases, contact with study authors, trial registers or other grey literature sources) with planned dates of coverage	6
Search strategy	10	Present draft of search strategy to be used for at least one electronic database, including planned limits, such that it could be repeated	6

For peer review only

Study records:			
Data management	11a	Describe the mechanism(s) that will be used to manage records and data throughout the review	8-10
Selection process	11b	State the process that will be used for selecting studies (such as two independent reviewers) through each phase of the review (that is, screening, eligibility and inclusion in meta-analysis)	7-8
Data collection process	11c	Describe planned method of extracting data from reports (such as piloting forms, done independently, in duplicate), any processes for obtaining and confirming data from investigators	7-8
Data items	12	List and define all variables for which data will be sought (such as PICO items, funding sources), any pre-planned data assumptions and simplifications	6-7
Outcomes and prioritization	13	List and define all outcomes for which data will be sought, including prioritization of main and additional outcomes, with rationale	8-10
Risk of bias in individual studies	14	Describe anticipated methods for assessing risk of bias of individual studies, including whether this will be done at the outcome or study level, or both; state how this information will be used in data synthesis	8-10
Data synthesis	15a	Describe criteria under which study data will be quantitatively synthesised	8-10
	15b	If data are appropriate for quantitative synthesis, describe planned summary measures, methods of handling data and methods of combining data from studies, including any planned exploration of consistency (such as I^2, Kendall's τ)	8-10
	15c	Describe any proposed additional analyses (such as sensitivity or subgroup analyses, meta-regression)	10
	15d	If quantitative synthesis is not appropriate, describe the type of summary planned	N/A
Meta-bias(es)	16	Specify any planned assessment of meta-bias(es) (such as publication bias across studies, selective reporting within studies)	10
Confidence in cumulative evidence	17	Describe how the strength of the body of evidence will be assessed (such as GRADE)	8

*** It is strongly recommended that this checklist be read in conjunction with the PRISMA-P Explanation and Elaboration (cite when available) for important clarification on the items. Amendments to a review protocol should be tracked and dated. The copyright for PRISMA-P (including checklist) is held by the PRISMA-P Group and is distributed under a Creative Commons Attribution Licence 4.0.**

From: Shamseer L, Moher D, Clarke M, Ghersi D, Liberati A, Petticrew M, Shekelle P, Stewart L, PRISMA-P Group. Preferred reporting items for systematic review and meta-analysis protocols (PRISMA-P) 2015: elaboration and explanation. BMJ. 2015 Jan 2;349(jan02 1):g7647.

For peer review only

BMJ Open

The association between quantitative sensory testing and pain or disability in paediatric and young adult chronic pain: Protocol for a systematic review and meta-analysis

Journal:	*BMJ Open*
Manuscript ID	bmjopen-2019-031861.R1
Article Type:	Protocol
Date Submitted by the Author:	07-Aug-2019
Complete List of Authors:	Schoth, Daniel; University of Southampton, School of Psychology<br>Blankenburg, Markus; University of Witten/Herdecke, Faculty of Health<br>Wager, Julia; Universitat Witten/Herdecke,<br>Broadbent, Philippa; University of Southampton, School of Psychology<br>Zhang, Jin; University of Southampton Faculty of Engineering and the Environment, School of Psychology<br>Zernikow, Boris; Universität Witten/Herdecke<br>Liossi, Christina; University of Southampton, School of Psychology
**Primary Subject Heading**:	Paediatrics
Secondary Subject Heading:	Research methods
Keywords:	Quantitative sensory testing, QST, Paediatric chronic pain, Disability, Systematic review, Meta-analysis

SCHOLARONE™
Manuscripts

The association between quantitative sensory testing and pain or disability in paediatric and young adult chronic pain: Protocol for a systematic review and meta-analysis

Schoth D. E.[1], Blankenburg M.[2,3], Wager J.[4,5], Broadbent P[1]., Zhang J[1]., Zernikow B.[4,5], and Liossi C.[1,2]

[1]Pain Research Laboratory, School of Psychology, University of Southampton, Highfield, Southampton, UK; [2] Department for Pediatric Neurology, Child Pain Center Baden-Württemberg, Klinikum Stuttgart, Germany; [3] Witten/Herdecke University, Faculty of Health, Witten, Germany; [4] German Paediatric Pain Centre, Children's and Adolescents' Hospital Datteln, Germany; [5] Department of Children's Pain Therapy and Paediatric Palliative Care, Witten/Herdecke University, Faculty of Health, School of Medicine, Witten, Germany; [6] Great Ormond Street Hospital for Children NHS Foundation trust, London, UK.

Corresponding author: Prof. Christina Liossi, Pain Research Laboratory, Department of Psychology, University of Southampton, Highfield, Southampton, SO17 1BJ, UK. E-mail: C.Liossi@soton.ac.uk, Tel: 0044 (0)238059 4645.

For peer review only - http://bmjopen.bmj.com/site/about/guidelines.xhtml

Abstract

Introduction. This protocol describes the objective and methods of a systematic review of the association between quantitative sensory testing (QST) measures and pain intensity or disability in paediatric chronic pain (PCP). The review will also assess whether the relationship strength is moderated by variables related to the QST method and pain condition; the use of QST in PCP (modalities, outcome measures and anatomical test sites as well as differentiating between pain mechanisms (e.g., neuropathic vs. nociceptive) and in selecting analgesics); the reliability of QST across the paediatric age range; the ability of QST to differentiate patients with chronic pain from healthy controls; and differences between anatomical test sites. **Methods and analysis.** MEDLINE, PsycINFO , CINHAL, Web of Science, Scopus, Cochrane Library and OpenGrey will be searched. English language studies will be eligible if they; recruit a sample aged 6 to 24 (inclusive) with chronic pain, including primary and secondary pain; apply at least one of the following QST modalities: chemical, electrical, mechanical (sub-groups include pressure, punctate/brush, vibratory) or thermal stimulus, to measure perception of noxious or innocuous stimuli applied to skin, muscle or joint; use a testing protocol to control for stimulus properties: modality, anatomical site, intensity, duration, and sequence. Following title and abstract screening, the full texts of relevant records will be independently assessed by two reviewers. For eligible studies, one reviewer will extract study characteristics and data, and another will check for accuracy. Both will undertake independent quality assessments using Appraisal Tool for Cross Sectional Studies (AXIS). A qualitative synthesis will be presented with discussion centred around different QST modalities. Where eligible data permit, meta-analyses will be performed separately for different QST modalities using Comprehensive Meta-Analysis. **Ethics and dissemination.** Review findings will be reported in a peer-reviewed journal and presented at conferences. The study raises no ethical issues.

For peer review only - http://bmjopen.bmj.com/site/about/guidelines.xhtml

Article Summary

Strengths and Limitations of this Study

- This systematic review can give us a greater understanding of the association between quantitative sensory testing (QST) (in accordance with either the DFNS (German research network on Neuropathic Pain) protocol or other protocols) and pain intensity or disability in paediatric chronic pain and could inform the use of QST in clinical practice
- This systematic review will follow robust guidelines and the quality of included articles will be assessed using validated tools
- The heterogeneity of the included studies, which may use different quantitative sensory measures across different chronic pain conditions and age range, could limit the overall data synthesis

Keywords

Quantitative sensory testing; QST; Pediatric chronic pain; Disability; Systematic review; Meta-analysis

For peer review only

Introduction

Paediatric chronic pain (PCP) is defined as persistent or recurring pain of any aetiology lasting for longer than three months.[1 2] A cross-sectional study of 561 schoolchildren aged 8 to 16 (mean age 11.89) showed a PCP prevalence of 37.3%, of which 29.3% reported pain in two or more sites.[1] Prevalence rates do vary across reports and conditions however, as demonstrated in a systematic review of 41 articles (e.g., 4% – 40% for musculoskeletal pain, 8% – 83% for headache, and 4% – 53% for abdominal pain) which may be due, in part, to inconsistent definitions of chronic pain adopted by individual studies [3]. An analysis of the 2016 National Survey of Children's Health in the United States estimated a population prevalence of PCP to be 6% based on a sample of 43,712 children aged 0 to 17 years.[4]

Despite variations in precise prevalence estimates, PCP is a significant clinical problem worldwide[5], and can have enormous impacts on the child's quality of life.[6] Compared to their healthy peers children with chronic pain report substantially worse quality of life in physical, emotional, social, and school functioning[7], and frequently experience disordered sleep.[8] A purely biomedical model of PCP is outdated, and instead the severity, impact and experience of chronic pain is now recognised to be shaped by a complex interaction of biological, psychological and social factors.[9] Multimodal interdisciplinary interventions are therefore essential in the treatment of severe PCP.[10 11] A recent review of 21 studies highlights the effectiveness of interdisciplinary interventions in PCP management. Patients receiving interdisciplinary interventions reported significant improvements pre- to post-intervention in pain intensity, functional disability, anxiety, depression, catastrophizing, school attendance, school functioning and pain acceptance, and significantly lower pain intensity 0-1 month post-intervention compared to patients randomised to control groups.[12]

Quantitative sensory testing (QST) refers to a range of non-invasive techniques for exploring somatosensory processing, including positive and negative sensory phenomena.[13 14] QST is psychophysical in nature, relying on the individual's subjective report to a standardized stimulus.[13 15] QST might provide unique information about the functional status of the somatosensory system and

For peer review only - http://bmjopen.bmj.com/site/about/guidelines.xhtml

therefore better guide pain treatment. Although QST is commonly used in adult populations, QST is also considered feasible, reliable and reproducible in children, including children as young as four years old when using thermal stimuli.[16] In recent years a small but growing number of published studies have explored the use of QST in PCP populations, although calls have been made to expand this further.[17] Similar to other psychophysical methods, and to enable the comparison to published normative values and between different QST labs as well as follow-up examinations, QST requires the active participation of the young patient, a standardized assessment using standardized instructions and calibrated stimuli applied by highly trained investigators. A number of protocols are followed to this end with the protocol proposed by the German Research Network on Neuropathic Pain (DFNS) the most well developed. DFNS includes examination of warm and cold detection and pain thresholds including the assessment of paradoxical heat sensation during alternating temperature stimuli (using a thermostat), mechanical detection thresholds for light touch (using a set of von Frey filaments), and for vibration (using a Rydel-Seifter tuning fork), mechanical pain thresholds for pinprick (using pinprick stimulators of different intensities) and for blunt pressure (using a pressure algometer) as well as a stimulus-response function for mechanical pain sensitivity to pinprick stimuli, dynamic mechanical allodynia for the stimuli of different intensities (using a cotton wool, a Q tip and a standardized brush) and temporal summation for pinprick stimuli.[18]

The primary objective of this systematic review and meta-analysis is to examine the relationship between established QST measures and pain intensity or disability in PCP. The second objective is to assess whether the strength of the relationship is moderated by variables related to the QST method and pain condition. Furthermore, this systematic review and meta-analysis will examine: [1] the use of QST in PCP (modalities, outcome measures and anatomical test sites differentiating between pain mechanisms (e.g., neuropathic vs nociceptive) and in selecting analgesics; [2] the reliability of QST across the paediatric age range; [3] the ability of QST to differentiate patients with chronic pain from healthy controls, and [4] differences between anatomical test sites.

For peer review only - http://bmjopen.bmj.com/site/about/guidelines.xhtml

Methods

This protocol was developed in line with PRISMA-P guidelines[19 20] and is registered with PROSPERO (CRD42019134069), an international register of systematic reviews. Any amendments to the protocol will be recorded on PROSPERO.

Literature Search

We will search the following electronic bibliographic databases: MEDLINE, PsycINFO, CINHAL (title, abstract), Web of Science (title), Scopus (article title, abstract, keywords), and Cochrane Library (title, abstract, keywords). A search of non-traditional publications, commonly known as 'grey literature', will be conducted using Open Grey. All databases will be searched from database inception to the date of search. Only studies published in the English language will be included. The reference lists of all eligible papers will be hand searched for relevant studies. The search strategy will include three concept blocks pertaining to the type of pain (*pain**, *ache**, *abdominal pain*, *arthritis*, *headache**, *musculoskeletal pain*, *fibromyalgia*), study population (*boy**, *girl**, *child**, *teenager**, *youth**, *adolescen**, *young**, *schoolchild**, *school child**, *juvenil**, *paediatric*, *pediatric*), and quantitative sensory testing (*quantitative sensory test**, *sensory test**, *QST*, *threshold*, *tolerance*, *pressure*, *electrical*, *warm*, *heat*, *hot*, *cool*, *cold*, *mechanical*, *hyperalgesia*, *hyperaesthesia*, *allodynia*, *sensitisation*, *sensitivity*). [Note: the asterisk is used as a truncation command to search the root word along with any endings, for example *child** would retrieve both *child* and *children*].

Inclusion Criteria

Studies will be eligible for inclusion if they meet the following criteria:

1) Available in the English language.
2) Recruits a sample aged 6 to 24 (inclusive) with chronic pain, including primary pain (i.e., pain in one or more anatomic regions that has lasted for longer than 3 months and cannot be better explained by any other chronic pain condition) and secondary pain (i.e., pain that has lasted

For peer review only - http://bmjopen.bmj.com/site/about/guidelines.xhtml

longer than 3 months that is symptomatic of another health condition).[21] In cases where the sample age range includes individuals older than 24 years of age, the record will only be eligible for inclusion if separate data is presented for the age group of interest to the present review. While the adolescent age range typically extends to 18 years, the transition period from childhood to adulthood is now argued to occupy a greater portion of the life course than before, and therefore an upper age limit of 24 years corresponds more closely to adolescent growth and popular understandings of this life phase.[22]

3) Applies at least one of the following QST modalities: chemical, electrical, mechanical (sub-groups will include pressure, punctate/brush, and vibratory) or thermal stimulus, in order to measure perception of noxious or innocuous stimuli applied to skin, muscle or joint.
4) Uses a QST testing protocol that allows reliable replication of the procedure followed and which controls for stimulus properties: modality, anatomical site, intensity, duration, and sequence.

Study exclusion criteria for this review are:

1) The use of an invasive form of QST only (e.g., rectal barostat).
2) Examination of pain modulation only.
3) Providing correlations between pain intensity/disability and QST composite scores (i.e., local and remote site combined) only.

QST is defined as a method that quantifies the magnitude of physical stimuli (e.g., pressure, heat, cold, vibration, electrical current) that is required to determine a specific perception (i.e., threshold, tolerance, temporal summation, magnitude rating, wind-up, limen). For inclusion in this systematic review, the application of the physical stimulus will have to be standardized and the physical stimulus will have to be expressed in quantitative terms (e.g., pressure: kg/cm^2; heat/cold: $^\circ$C). The evoked sensory and pain perception will have to be reported subjectively by the participant (e.g., yes/no for pin prick threshold) and quantitatively (e.g., pressure: kg/cm^2; heat/cold: $^\circ$C; intensity ratings using VAS, NRS or other validated scales). Correlation coefficients (Pearson's r or Spearman's q) for the

For peer review only - http://bmjopen.bmj.com/site/about/guidelines.xhtml
For peer review only

relationship between QST measured locally and/or at a remote site and pain intensity/disability will have to be reported or could be calculated from the data reported in the study or from data obtained from the study authors.

Data Extraction

The titles and abstracts of retrieved records will be transferred into the digital reference manager Endnote X8. Titles will be scanned for duplication using an automated search engine within Endnote, and any duplicates will be removed. Records will then be screened by two review team members independently to identify studies that potentially meet the inclusion criteria. The full texts of potentially relevant records will be retrieved, which will be independently assessed for eligibility by the same two review team members. Any disagreement between reviewers over study eligibility will be resolved through discussion with a third review author. Data will be extracted using a pre-piloted template. Extracted information will include the country in which the research was conducted; demographics and medical characteristics of PCP patients; demographics of healthy controls (where applicable); QST protocol details; the training of the QST administrators; the outcome measures recorded; and QST and other outcome results. For eligible studies, one reviewer will perform extraction of study characteristics and data, and another will check for accuracy (with a third review author where necessary). If information is missing or unclear, the corresponding author for the study in question will be contacted.

For peer review only

Study Quality Assessment

Two review authors will independently assess study quality using Appraisal Tool for Cross Sectional Studies (AXIS).[23] AXIS features 20 questions which are answered as either 'yes', 'no' or 'don't know', along with spaces for comments on each judgement provided. Although similar to the STROBE statement (a 22-item checking for use with observational (cohort, cross-sectional, case-control) studies[24]), AXIS provides an assessment of both study design and risk of bias. Interrater reliability between the two reviewers will be calculated using Cohen's Kappa, where a Kappa value of .80 or above is considered a strong level of agreement and indicates high interrater reliability.[25]

For peer review only - http://bmjopen.bmj.com/site/about/guidelines.xhtml

Any disagreements between the review authors will be resolved by discussion, with involvement of a third review author where necessary.

Statistical Analysis

Meta-analysis will be performed to address the study objectives where data from two or more eligible studies is available.[26] Data will be grouped into clinically meaningful diagnostic categories (e.g., abdominal pain, migraine, tension-type headache, fibromyalgia, arthritis) and analysed separately. Due to developmental and sex differences in somatosensory perception[27 28] data will be grouped and analysed separately where possible for males and females, and by appropriate age groups. Regarding the latter we anticipate we will analyse two groups (i) 6-20 and (ii) 21 and over, unless there is enough data and we are able to follow and modify the groupings suggested by Blankenburg and colleagues[27] (i.e., younger children (6–8 years), older children (9–12 years), younger adolescents (13–16 years) and older adolescents (17-24 years)). Data will also be grouped into local (i.e., the site of pain) and non-local (i.e., a non-painful site) categories. Analyses will be conducted separately by type of stimuli (i.e., pressure, mechanical, heat, cold, vibration, electrical, chemical) due to different pathways mediating the nociceptive and pain experience.

All statistical analyses will be performed using Comprehensive Meta-Analysis (CMA, version 3.0[29]) and Power and Sample Size Calculations 3.0.43 software. CMA converts correlation coefficients to Fisher's z scores, with all analyses performed using these transformed values.[30] Similar to a recent review of QST in spinal pain[31] Pearson's r or Spearman's rank coefficient will be synthesized together. Unlike Pearson's product-moment correlation coefficient, which requires the assumption that the relationship between the variables is linear and variables to be measured on interval scales, Spearman's rank correlation coefficient is a nonparametric (distribution-free) rank statistic. It assesses how well an arbitrary monotonic function can describe a relationship between two variables, without making any assumptions about the frequency distribution of the variables and can be used for variables measured at the ordinal level. We will use .25, .5, and .75 as cut-off points to

For peer review only - http://bmjopen.bmj.com/site/about/guidelines.xhtml

interpret the strength of the relationship as little or zero (.00 to .25), fair (.26 to .50), moderate to good (.51 to .75), and good to excellent (above .75).[32]

Heterogeneity will be examined using Cochrane's Q test and the I^2 statistic. With Cochrane's Q, a significant result is indicative of heterogeneity. The I^2 statistic describes the percentage of variability in effect estimates due to heterogeneity as opposed to sampling error [33]. The Cochrane Handbook provides the following rough guide for interpretation of the I^2 statistic: 0% to 40%: might not be important; 30% to 60%: may represent moderate heterogeneity; 50% to 90%: may represent substantial heterogeneity; 75% to 100%: considerable heterogeneity.[33] We will conduct sensitivity analyses where appropriate to verify the robustness of the study conclusions, and for more than 10 studies will will use funnel plots to detect potential reporting biases and small-study effects. Given sufficient availability of data, mixed effects meta-regression will be used to formally assess the effect of the potential categorical predictors (QST testing site, pain condition, pain type, type of pain induction stimulus) on the strength of the relationship between pain threshold and pain intensity/disability. The effect of each categorical predictor will be tested in a separate meta-regression.

The ability of QST to detect somatosensory differences between young people with chronic pain and healthy controls will be assessed using standardised mean differences (SMDs). The standardised mean difference will be calculated as the mean difference between groups divided by the standard deviation of the specific QST measure for all participants pooled across both groups. The standardised mean difference expresses the difference between groups in QST measures as multiples of the observed standard deviation. The standardised mean difference is a ratio, with numerator and denominator in the same units as the original measurement, therefore has no units, does not depend on the original measurement scale, and allows a direct comparison of the effect across studies that used different QST measures. By convention, a SMD or $d = 0.2$ is considered to be small, $d = 0.5$ moderate and $d = 0.8$ large in size.[34] Each study will be screened for intraclass correlation coefficients (ICCs)

For peer review only - http://bmjopen.bmj.com/site/about/guidelines.xhtml

describing the test-retest reliability of QST. The reliability of the measurement across test occasions will be rated excellent if the ICC > .75, adequate if .40 – .74, and poor if < .40.[35]

Patient and public involvement

Consultation with young people, parents and health care professionals has been used to determine their perception of the clinical utility of quantitative sensory testing in chronic pain. It is based on their perspectives that this systematic review was deemed timely and crucial to conduct to inform further research work and implementation of QST in routine clinical practice.

Discussion

This systematic review will be the first to investigate the association between QST and pain intensity or disability in paediatric chronic pain. The review findings will be used to inform our ongoing work to develop a core outcome set for specialist paediatric chronic pain services across the UK.

Ethics and dissemination

As this is a systematic review of published literature, ethical approval will not be sought. We will publish the protocol and our findings in peer-reviewed journals aimed at paediatric chronic pain clinicians and researchers as well as health commissioners. We will present our work at national and international meetings focused on paediatric chronic pain.

Acknowledgements

We would like to thank Holly Brown, Navin Down, Victoria Gibbons and Olivia Ranger (BSc Psychology students studying at the University of Southampton who undertook their 3rd year dissertation projects at the Pain Research Laboratory) for performing a scoping review of relevant literature that informed the development of this protocol.

Author Contributions

CL conceived the idea, planned and designed the study protocol. CL and DES planned the data extraction and statistical analysis and wrote the first draft; MB, JW, BZ, JZ and PB provided critical insights. All authors have approved and contributed to the final written manuscript.

For peer review only - http://bmjopen.bmj.com/site/about/guidelines.xhtml

Funding

This development of this protocol was supported by ESRC (Grant number: RES-000-22-4128). This funding source had no role in the design of this review and will not have any role during its execution, analyses, interpretation of the data, or decision to submit results.

Competing Interests

None declared.

For peer review only

References

1. Huguet A, Miró J. The severity of chronic pediatric pain: an epidemiological study. J Pain 2008;**9**(3):226-36.
2. Merskey H, & Bogduk, N. *Classification of chronic pain: Descriptions of chronic pain syndromes and definitions of pain terms*. Seattle: IASP Press 1994.
3. King S, Chambers CT, Huguet A, et al. The epidemiology of chronic pain in children and adolescents revisited: a systematic review. Pain 2011;**152**(12):2729-38 doi: 10.1016/j.pain.2011.07.016.
4. Tumin D, Drees D, Miller R, et al. Health Care Utilization and Costs Associated With Pediatric Chronic Pain. J Pain 2018;**19**(9):973-82 doi: 10.1016/j.jpain.2018.03.012.
5. Miró J, McGrath PJ, Finley GA, et al. Pediatric chronic pain programs: current and ideal practice. Pain Reports 2017;**2**(5):e613 doi: 10.1097/PR9.0000000000000613.
6. Rolfe PM. Paediatric chronic pain. Anaesthesia & Intensive Care Medicine 2016;**17**(11):531-35.
7. Varni JW, Limbers CA, Burwinkle TM. Impaired health-related quality of life in children and adolescents with chronic conditions: a comparative analysis of 10 disease clusters and 33 disease categories/severities utilizing the PedsQL 4.0 Generic Core Scales. Health Qual Life Outcomes 2007;**5**:43 doi: 10.1186/1477-7525-5-43.
8. Evans S, Djilas V, Seidman LC, et al. Sleep Quality, Affect, Pain, and Disability in Children With Chronic Pain: Is Affect a Mediator or Moderator? J Pain 2017;**18**(9):1087-95 doi: 10.1016/j.jpain.2017.04.007.
9. Liossi C, Howard RF. Pediatric chronic pain: biopsychosocial assessment and formulation. Pediatrics 2016;**138**(5):e20160331.
10. Rajapakse D, Liossi C, Howard RF. Presentation and management of chronic pain. Arch Dis Child 2014;**99**(5):474-80 doi: 10.1136/archdischild-2013-304207
11. Odell S, Logan DE. Pediatric pain management: the multidisciplinary approach. J Pain Res 2016;**6**:785–90. doi: 10.2147/JPR.S37434.

For peer review only - http://bmjopen.bmj.com/site/about/guidelines.xhtml

12. Liossi C, Johnstone L, Lilley S, et al. Interdisciplinary interventions for pediatric chronic pain: A systematic review and meta-analysis. Br J Anaesth in press.

13. Yarnitsky D. Quantitative Sensory Testing. Muscle Nerve 1997;**20**(2):198 - 204.

14. Yarnitsky D, Pud D. Quantitative sensory testing. In: Binnie CD, Cooper R, Mauguiere F, et al., eds. Clinical neurophysiology: EMG, nerve conduction and evoked potentials, vol1. Amsterdam: Elsevier, 2004:305 - 28.

15. Zaslansky R, Yarnitsky D. Clinical applications of quantitative sensory testing (QST). J Neurol Sci 1998;**153**(2):215-38 doi: https://doi.org/10.1016/S0022-510X(97)00293-1.

16. Hilz MJ, Glorius SE, Schweibold G, et al. Quantitative thermal perception testing in preschool children. Muscle & Nerve: Official Journal of the American Association of Electrodiagnostic Medicine 1996;**19**(3):381-83.

17. Tham SW, Palermo TM, Holley AL, et al. A population-based study of quantitative sensory testing in adolescents with and without chronic pain. Pain 2016;**157**(12):2807-15 doi: 10.1097/j.pain.0000000000000716.

18. Rolke R, Baron R, Maier C, et al. Quantitative sensory testing in the German Research Network on Neuropathic Pain (DFNS): Standardized protocol and reference values. Pain 2006;**123**(3):231-43.

19. Moher D, Shamseer L, Clarke M, et al. Preferred reporting items for systematic review and meta-analysis protocols (PRISMA-P) 2015 statement. Systematic Reviews 2015;**4**(1):1.

20. Shamseer L, Moher D, Clarke M, et al. Preferred reporting items for systematic review and meta-analysis protocols (PRISMA-P) 2015: elaboration and explanation. BMJ 2015;**349**:g7647 doi: 10.1136/bmj.g7647

21. Treede R-D, Rief W, Barke A, et al. A classification of chronic pain for ICD-11. Pain 2015;**156**(6):1003 doi: 0.1097/j.pain.0000000000000160.

22. Sawyer SM, Azzopardi PS, Wickremarathne D, et al. The age of adolescence. The Lancet: Child and Adolescent Health 2018;**2**(3):223 - 28 doi: 10.1016/S2352-4642(18)30022-1.

For peer review only - http://bmjopen.bmj.com/site/about/guidelines.xhtml

23. Downes MJ, Brennan ML, Williams HC, et al. Development of a critical appraisal tool to assess the quality of cross-sectional studies (AXIS). BMJ Open 2016;**6**(12):e011458 doi: 10.1136/bmjopen-2016-011458.

24. Von Elm E, Altman DG, Egger M, et al. The Strengthening the Reporting of Observational Studies in Epidemiology (STROBE) statement: guidelines for reporting observational studies. PLoS Med 2007;**4**(10):e296.

25. McHugh ML. Interrater reliability: The kappa statistic. Biochemia Medica 2012;**22**(3):276-82.

26. Higgins JP, Green S. *Cochrane handbook for systematic reviews of interventions*: John Wiley & Sons, 2011.

27. Blankenburg M, Boekens H, Hechler T, et al. Reference values for quantitative sensory testing in children and adolescents: Developmental and gender differences of somatosensory perception. Pain 2010;**149**(1):76-88 doi: http://dx.doi.org/10.1016/j.pain.2010.01.011.

28. Blankenburg M, Meyer D, Hirschfeld G, et al. Developmental and sex differences in somatosensory perception—a systematic comparison of 7- versus 14-year-olds using quantitative sensory testing. . Pain 2011;**152**(11):2625 - 31 doi: 10.1016/j.pain.2011.08.007.

29. Comprehensive Meta-Analysis Version 3 [program]. Englewood, New Jersey: Biostat, 2013.

30. Borenstein M, Hedges LV, Higgins J, et al. *Introduction to meta-analysis*. Cornwell: Wiley Online Library, 2009.

31. Hübscher M, Moloney N, Leaver A, et al. Relationship between quantitative sensory testing and pain or disability in people with spinal pain—a systematic review and meta-analysis. PAIN® 2013;**154**(9):1497-504.

32. Portney LG. *Foundations of Clinical Research: Applications to Practice 3th (third) Edition*. New Jersey: Pearson Education, 2009.

33. Higgins JPT, Green S. *Cochrane Handbook for Systematic Reviews of Interventions*: The Cochrane Collaboration, 2008.

34. Cohen J. *Statistical power analysis for the behavioral sciences, 2nd edition* Hillsdale: Erlbaum, 1988.

For peer review only - http://bmjopen.bmj.com/site/about/guidelines.xhtml

35. Fleiss JL, Levin B, Paik MC. *Statistical Methods for Rates and Proportions*: John Wiley & Sons, 2013.

For peer review only

For peer review only - http://bmjopen.bmj.com/site/about/guidelines.xhtml

PRISMA-P (Preferred Reporting Items for Systematic review and Meta-Analysis Protocols) 2015 checklist: recommended items to address in a systematic review protocol*

Section and topic	Item No	Checklist item	Page
**ADMINISTRATIVE INFORMATION**			
Title:			
Identification	1a	Identify the report as a protocol of a systematic review	1
Update	1b	If the protocol is for an update of a previous systematic review, identify as such	N/A
Registration	2	If registered, provide the name of the registry (such as PROSPERO) and registration number	6
Authors:			
Contact	3a	Provide name, institutional affiliation, e-mail address of all protocol authors; provide physical mailing address of corresponding author	1
Contributions	3b	Describe contributions of protocol authors and identify the guarantor of the review	11
Amendments	4	If the protocol represents an amendment of a previously completed or published protocol, identify as such and list changes; otherwise, state plan for documenting important protocol amendments	N/A
Support:			
Sources	5a	Indicate sources of financial or other support for the review	12
Sponsor	5b	Provide name for the review funder and/or sponsor	12
Role of sponsor or funder	5c	Describe roles of funder(s), sponsor(s), and/or institution(s), if any, in developing the protocol	12
**INTRODUCTION**			
Rationale	6	Describe the rationale for the review in the context of what is already known	4-5
Objectives	7	Provide an explicit statement of the question(s) the review will address with reference to participants, interventions, comparators, and outcomes (PICO)	5
**METHODS**			
Eligibility criteria	8	Specify the study characteristics (such as PICO, study design, setting, time frame) and report characteristics (such as years considered, language, publication status) to be used as criteria for eligibility for the review	6-8
Information sources	9	Describe all intended information sources (such as electronic databases, contact with study authors, trial registers or other grey literature sources) with planned dates of coverage	6
Search strategy	10	Present draft of search strategy to be used for at least one electronic database, including planned limits, such that it could be repeated	6

For peer review only

Study records:			
Data management	11a	Describe the mechanism(s) that will be used to manage records and data throughout the review	8-10
Selection process	11b	State the process that will be used for selecting studies (such as two independent reviewers) through each phase of the review (that is, screening, eligibility and inclusion in meta-analysis)	8
Data collection process	11c	Describe planned method of extracting data from reports (such as piloting forms, done independently, in duplicate), any processes for obtaining and confirming data from investigators	8
Data items	12	List and define all variables for which data will be sought (such as PICO items, funding sources), any pre-planned data assumptions and simplifications	6-8
Outcomes and prioritization	13	List and define all outcomes for which data will be sought, including prioritization of main and additional outcomes, with rationale	8-10
Risk of bias in individual studies	14	Describe anticipated methods for assessing risk of bias of individual studies, including whether this will be done at the outcome or study level, or both; state how this information will be used in data synthesis	8-10
Data synthesis	15a	Describe criteria under which study data will be quantitatively synthesised	8-10
	15b	If data are appropriate for quantitative synthesis, describe planned summary measures, methods of handling data and methods of combining data from studies, including any planned exploration of consistency (such as I^2, Kendall's τ)	8-11
	15c	Describe any proposed additional analyses (such as sensitivity or subgroup analyses, meta-regression)	10
	15d	If quantitative synthesis is not appropriate, describe the type of summary planned	N/A
Meta-bias(es)	16	Specify any planned assessment of meta-bias(es) (such as publication bias across studies, selective reporting within studies)	10
Confidence in cumulative evidence	17	Describe how the strength of the body of evidence will be assessed (such as GRADE)	8-9

*** It is strongly recommended that this checklist be read in conjunction with the PRISMA-P Explanation and Elaboration (cite when available) for important clarification on the items. Amendments to a review protocol should be tracked and dated. The copyright for PRISMA-P (including checklist) is held by the PRISMA-P Group and is distributed under a Creative Commons Attribution Licence 4.0.**

From: Shamseer L, Moher D, Clarke M, Ghersi D, Liberati A, Petticrew M, Shekelle P, Stewart L, PRISMA-P Group. Preferred reporting items for systematic review and meta-analysis protocols (PRISMA-P) 2015: elaboration and explanation. BMJ. 2015 Jan 2;349(jan02 1):g7647.

For peer review only

The association between quantitative sensory testing and pain or disability in paediatric and young adult chronic pain: Protocol for a systematic review and meta-analysis

Journal:	*BMJ Open*
Manuscript ID	bmjopen-2019-031861.R2
Article Type:	Protocol
Date Submitted by the Author:	03-Sep-2019
Complete List of Authors:	Schoth, Daniel; University of Southampton, School of Psychology<br>Blankenburg, Markus; University of Witten/Herdecke, Faculty of Health<br>Wager, Julia; Universitat Witten/Herdecke,<br>Broadbent, Philippa; University of Southampton, School of Psychology<br>Zhang, Jin; University of Southampton Faculty of Engineering and the Environment, School of Psychology<br>Zernikow, Boris; Universität Witten/Herdecke<br>Liossi, Christina; University of Southampton, School of Psychology
**Primary Subject Heading**:	Paediatrics
Secondary Subject Heading:	Research methods
Keywords:	Quantitative sensory testing, QST, Paediatric chronic pain, Disability, Systematic review, Meta-analysis

SCHOLARONE™
Manuscripts

The association between quantitative sensory testing and pain or disability in paediatric and young adult chronic pain: Protocol for a systematic review and meta-analysis

Schoth D. E.[1], Blankenburg M.[2,3], Wager J.[4,5], Broadbent P[1]., Zhang J[1]., Zernikow B.[4,5], and Liossi C.[1,2]

[1]Pain Research Laboratory, School of Psychology, University of Southampton, Highfield, Southampton, UK; [2] Department for Pediatric Neurology, Child Pain Center Baden-Württemberg, Klinikum Stuttgart, Germany; [3] Witten/Herdecke University, Faculty of Health, Witten, Germany; [4] German Paediatric Pain Centre, Children's and Adolescents' Hospital Datteln, Germany; [5] Department of Children's Pain Therapy and Paediatric Palliative Care, Witten/Herdecke University, Faculty of Health, School of Medicine, Witten, Germany; [6] Great Ormond Street Hospital for Children NHS Foundation trust, London, UK.

Corresponding author: Prof. Christina Liossi, Pain Research Laboratory, Department of Psychology, University of Southampton, Highfield, Southampton, SO17 1BJ, UK. E-mail: C.Liossi@soton.ac.uk, Tel: 0044 (0)238059 4645.

For peer review only

Abstract

Introduction. This protocol describes the objective and methods of a systematic review of the association between quantitative sensory testing (QST) measures and pain intensity or disability in paediatric chronic pain (PCP). The review will also assess whether the relationship strength is moderated by variables related to the QST method and pain condition; the use of QST in PCP (modalities, outcome measures and anatomical test sites as well as differentiating between pain mechanisms (e.g., neuropathic vs. nociceptive) and in selecting analgesics); the reliability of QST across the paediatric age range; the ability of QST to differentiate patients with chronic pain from healthy controls; and differences between anatomical test sites. **Methods and analysis.** MEDLINE, PsycINFO , CINHAL, Web of Science, Scopus, Cochrane Library and OpenGrey will be searched. English language studies will be eligible if they; recruit a sample aged 6 to 24 (inclusive) with chronic pain, including primary and secondary pain; apply at least one of the following QST modalities: chemical, electrical, mechanical (sub-groups include pressure, punctate/brush, vibratory) or thermal stimulus, to measure perception of noxious or innocuous stimuli applied to skin, muscle or joint; use a testing protocol to control for stimulus properties: modality, anatomical site, intensity, duration, and sequence. Following title and abstract screening, the full texts of relevant records will be independently assessed by two reviewers. For eligible studies, one reviewer will extract study characteristics and data, and another will check for accuracy. Both will undertake independent quality assessments using Appraisal Tool for Cross Sectional Studies (AXIS). A qualitative synthesis will be presented with discussion centred around different QST modalities. Where eligible data permit, meta-analyses will be performed separately for different QST modalities using Comprehensive Meta-Analysis. **Ethics and dissemination.** Review findings will be reported in a peer-reviewed journal and presented at conferences. The study raises no ethical issues.

For peer review only - http://bmjopen.bmj.com/site/about/guidelines.xhtml

Article Summary

Strengths and Limitations of this Study

- This systematic review can give us a greater understanding of the association between quantitative sensory testing (QST) (in accordance with either the DFNS (German research network on Neuropathic Pain) protocol or other protocols) and pain intensity or disability in paediatric chronic pain and could inform the use of QST in clinical practice
- This systematic review will follow robust guidelines and the quality of included articles will be assessed using validated tools
- The heterogeneity of the included studies, which may use different quantitative sensory measures across different chronic pain conditions and age range, could limit the overall data synthesis

Keywords

Quantitative sensory testing; QST; Pediatric chronic pain; Disability; Systematic review; Meta-analysis

For peer review only

Introduction

Paediatric chronic pain (PCP) is defined as persistent or recurring pain of any aetiology lasting for longer than three months.[1 2] A cross-sectional study of 561 schoolchildren aged 8 to 16 (mean age 11.89) showed a PCP prevalence of 37.3%, of which 29.3% reported pain in two or more sites.[1] Prevalence rates do vary across reports and conditions however, as demonstrated in a systematic review of 41 articles (e.g., 4% – 40% for musculoskeletal pain, 8% – 83% for headache, and 4% – 53% for abdominal pain) which may be due, in part, to inconsistent definitions of chronic pain adopted by individual studies [3]. An analysis of the 2016 National Survey of Children's Health in the United States estimated a population prevalence of PCP to be 6% based on a sample of 43,712 children aged 0 to 17 years.[4]

Despite variations in precise prevalence estimates, PCP is a significant clinical problem worldwide[5], and can have enormous impacts on the child's quality of life.[6] Compared to their healthy peers children with chronic pain report substantially worse quality of life in physical, emotional, social, and school functioning[7], and frequently experience disordered sleep.[8] A purely biomedical model of PCP is outdated, and instead the severity, impact and experience of chronic pain is now recognised to be shaped by a complex interaction of biological, psychological and social factors.[9] Multimodal interdisciplinary interventions are therefore essential in the treatment of severe PCP.[10 11] A recent review of 21 studies highlights the effectiveness of interdisciplinary interventions in PCP management. Patients receiving interdisciplinary interventions reported significant improvements pre- to post-intervention in pain intensity, functional disability, anxiety, depression, catastrophizing, school attendance, school functioning and pain acceptance, and significantly lower pain intensity 0-1 month post-intervention compared to patients randomised to control groups.[12]

Quantitative sensory testing (QST) refers to a range of non-invasive techniques for exploring somatosensory processing, including positive and negative sensory phenomena.[13 14] QST is psychophysical in nature, relying on the individual's subjective report to a standardized stimulus.[13 15] QST might provide unique information about the functional status of the somatosensory system and

For peer review only - http://bmjopen.bmj.com/site/about/guidelines.xhtml

therefore better guide pain treatment. Although QST is commonly used in adult populations, QST is also considered feasible, reliable and reproducible in children, including children as young as four years old when using thermal stimuli.[16] In recent years a small but growing number of published studies have explored the use of QST in PCP populations, although calls have been made to expand this further.[17] Similar to other psychophysical methods, and to enable the comparison to published normative values and between different QST labs as well as follow-up examinations, QST requires the active participation of the young patient, a standardized assessment using standardized instructions and calibrated stimuli applied by highly trained investigators. A number of protocols are followed to this end with the protocol proposed by the German Research Network on Neuropathic Pain (DFNS) the most well developed. DFNS includes examination of warm and cold detection and pain thresholds including the assessment of paradoxical heat sensation during alternating temperature stimuli (using a thermostat), mechanical detection thresholds for light touch (using a set of von Frey filaments), and for vibration (using a Rydel-Seifter tuning fork), mechanical pain thresholds for pinprick (using pinprick stimulators of different intensities) and for blunt pressure (using a pressure algometer) as well as a stimulus-response function for mechanical pain sensitivity to pinprick stimuli, dynamic mechanical allodynia for the stimuli of different intensities (using a cotton wool, a Q tip and a standardized brush) and temporal summation for pinprick stimuli.[18]

The primary objective of this systematic review and meta-analysis is to examine the relationship between established QST measures and pain intensity or disability in PCP. The second objective is to assess whether the strength of the relationship is moderated by variables related to the QST method and pain condition. Furthermore, this systematic review and meta-analysis will examine: [1] the use of QST in PCP (modalities, outcome measures and anatomical test sites differentiating between pain mechanisms (e.g., neuropathic vs nociceptive) and in selecting analgesics; [2] the reliability of QST across the paediatric age range; [3] the ability of QST to differentiate patients with chronic pain from healthy controls, and [4] differences between anatomical test sites.

For peer review only - http://bmjopen.bmj.com/site/about/guidelines.xhtml

Methods

This protocol was developed in line with PRISMA-P guidelines[19 20] and is registered with PROSPERO (CRD42019134069), an international register of systematic reviews. Any amendments to the protocol will be recorded on PROSPERO.

Literature Search

We will search the following electronic bibliographic databases: MEDLINE, PsycINFO, CINHAL (title, abstract), Web of Science (title), Scopus (article title, abstract, keywords), and Cochrane Library (title, abstract, keywords). A search of non-traditional publications, commonly known as 'grey literature', will be conducted using Open Grey. All databases will be searched from database inception to the date of search. Only studies published in the English language will be included. The reference lists of all eligible papers will be hand searched for relevant studies. The search strategy will include three concept blocks pertaining to the type of pain (*pain**, *ache**, *abdominal pain*, *arthritis*, *headache**, *musculoskeletal pain*, *fibromyalgia*), study population (*boy**, *girl**, *child**, *teenager**, *youth**, *adolescen**, *young**, *schoolchild**, *school child**, *juvenil**, *paediatric*, *pediatric*), and quantitative sensory testing (*quantitative sensory test**, *sensory test**, *QST*, *threshold*, *tolerance*, *pressure*, *electrical*, *warm*, *heat*, *hot*, *cool*, *cold*, *mechanical*, *hyperalgesia*, *hyperaesthesia*, *allodynia*, *sensitisation*, *sensitivity*). [Note: the asterisk is used as a truncation command to search the root word along with any endings, for example *child** would retrieve both *child* and *children*].

Inclusion Criteria

Studies will be eligible for inclusion if they meet the following criteria:

1) Available in the English language.
2) Recruits a sample aged 6 to 24 (inclusive) with chronic pain, including primary pain (i.e., pain in one or more anatomic regions that has lasted for longer than 3 months and cannot be better explained by any other chronic pain condition) and secondary pain (i.e., pain that has lasted

For peer review only - http://bmjopen.bmj.com/site/about/guidelines.xhtml

longer than 3 months that is symptomatic of another health condition).[21] In cases where the sample age range includes individuals older than 24 years of age, the record will only be eligible for inclusion if separate data is presented for the age group of interest to the present review. While the adolescent age range typically extends to 18 years, the transition period from childhood to adulthood is now argued to occupy a greater portion of the life course than before, and therefore an upper age limit of 24 years corresponds more closely to adolescent growth and popular understandings of this life phase.[22]

3) Applies at least one of the following QST modalities: chemical, electrical, mechanical (sub-groups will include pressure, punctate/brush, and vibratory) or thermal stimulus, in order to measure perception of noxious or innocuous stimuli applied to skin, muscle or joint.
4) Uses a QST testing protocol that allows reliable replication of the procedure followed and which controls for stimulus properties: modality, anatomical site, intensity, duration, and sequence.

For peer review only

Study exclusion criteria for this review are:

1) The use of an invasive form of QST only (e.g., rectal barostat).
2) Examination of pain modulation only.
3) Providing correlations between pain intensity/disability and QST composite scores (i.e., local and remote site combined) only.

QST is defined as a method that quantifies the magnitude of physical stimuli (e.g., pressure, heat, cold, vibration, electrical current) that is required to determine a specific perception (i.e., threshold, tolerance, temporal summation, magnitude rating, wind-up, limen). For inclusion in this systematic review, the application of the physical stimulus will have to be standardized and the physical stimulus will have to be expressed in quantitative terms (e.g., pressure: kg/cm^2; heat/cold: ˚C). The evoked sensory and pain perception will have to be reported subjectively by the participant (e.g., yes/no for pin prick threshold) and quantitatively (e.g., pressure: kg/cm^2; heat/cold: ˚C; intensity ratings using VAS, NRS or other validated scales). Correlation coefficients (Pearson's *r* or Spearman's *q*) for the

relationship between QST measured locally and/or at a remote site and pain intensity/disability will have to be reported or could be calculated from the data reported in the study or from data obtained from the study authors.

Data Extraction

The titles and abstracts of retrieved records will be transferred into the digital reference manager Endnote X8. Titles will be scanned for duplication using an automated search engine within Endnote, and any duplicates will be removed. Records will then be screened by two review team members independently to identify studies that potentially meet the inclusion criteria (two independent reviewers were decided upon based on PRISMA guidance[23] and the helpful comments from an independent reviewer of this protocol manuscript). The full texts of potentially relevant records will be retrieved, which will be independently assessed for eligibility by the same two review team members. Any disagreement between reviewers over study eligibility will be resolved through discussion with a third review author. Data will be extracted using a pre-piloted template. Extracted information will include the country in which the research was conducted; demographics and medical characteristics of PCP patients; demographics of healthy controls (where applicable); QST protocol details; the training of the QST administrators; the outcome measures recorded; and QST and other outcome results. For eligible studies, one reviewer will perform extraction of study characteristics and data, and another will check for accuracy (with a third review author where necessary). If information is missing or unclear, the corresponding author for the study in question will be contacted.

Study Quality Assessment

Two review authors will independently assess study quality using Appraisal Tool for Cross Sectional Studies (AXIS).[24] AXIS features 20 questions which are answered as either 'yes', 'no' or 'don't know', along with spaces for comments on each judgement provided. Although similar to the STROBE statement (a 22-item checking for use with observational (cohort, cross-sectional, case-control) studies[25]), AXIS provides an assessment of both study design and risk of bias. Interrater reliability between the two reviewers will be calculated using Cohen's Kappa, where a Kappa value

For peer review only - http://bmjopen.bmj.com/site/about/guidelines.xhtml

of .80 or above is considered a strong level of agreement and indicates high interrater reliability.[26] Any disagreements between the review authors will be resolved by discussion, with involvement of a third review author where necessary.

Statistical Analysis

Meta-analysis will be performed to address the study objectives where data from two or more eligible studies is available.[27] Data will be grouped into clinically meaningful diagnostic categories (e.g., abdominal pain, migraine, tension-type headache, fibromyalgia, arthritis) and analysed separately. Due to developmental and sex differences in somatosensory perception[28 29] data will be grouped and analysed separately where possible for males and females, and by appropriate age groups. Regarding the latter we anticipate we will analyse two groups (i) 6-20 and (ii) 21 and over, unless there is enough data and we are able to follow and modify the groupings suggested by Blankenburg and colleagues[28] (i.e., younger children (6–8 years), older children (9–12 years), younger adolescents (13–16 years) and older adolescents (17-24 years)). Where data are not provided separately per age group (for example if the sample age ranges from 18 – 24), we will contact the study authors and request separate data. If separate data are not provided or available we will not be able to include the data in any relevant meta-analyses. Data will also be grouped into local (i.e., the site of pain) and non-local (i.e., a non-painful site) categories and will initially be analysed separately, although based on the suggestion of an independent reviewer will also perform an analysis of all sites combined where possible. Analyses will be conducted separately by type of stimuli (i.e., pressure, mechanical, heat, cold, vibration, electrical, chemical) due to different pathways mediating the nociceptive and pain experience.

All statistical analyses will be performed using Comprehensive Meta-Analysis (CMA, version 3.0[30]) and Power and Sample Size Calculations 3.0.43 software. CMA converts correlation coefficients to Fisher's *z* scores, with all analyses performed using these transformed values.[31] Similar to a recent review of QST in spinal pain[32] Pearson's *r* or Spearman's rank coefficient will be synthesized together. Unlike Pearson's product-moment correlation coefficient, which requires the

For peer review only

For peer review only - http://bmjopen.bmj.com/site/about/guidelines.xhtml

assumption that the relationship between the variables is linear and variables to be measured on interval scales, Spearman's rank correlation coefficient is a nonparametric (distribution-free) rank statistic. It assesses how well an arbitrary monotonic function can describe a relationship between two variables, without making any assumptions about the frequency distribution of the variables and can be used for variables measured at the ordinal level. We will use .25, .5, and .75 as cut-off points to interpret the strength of the relationship as little or zero (.00 to .25), fair (.26 to .50), moderate to good (.51 to .75), and good to excellent (above .75).[33]

Heterogeneity will be examined using Cochrane's Q test and the I^2 statistic. With Cochrane's Q, a significant result is indicative of heterogeneity. The I^2 statistic describes the percentage of variability in effect estimates due to heterogeneity as opposed to sampling error [34]. The Cochrane Handbook provides the following rough guide for interpretation of the I^2 statistic: 0% to 40%: might not be important; 30% to 60%: may represent moderate heterogeneity; 50% to 90%: may represent substantial heterogeneity; 75% to 100%: considerable heterogeneity.[34] We will conduct sensitivity analyses where appropriate to verify the robustness of the study conclusions, and for more than 10 studies will will use funnel plots to detect potential reporting biases and small-study effects. Given sufficient availability of data, mixed effects meta-regression will be used to formally assess the effect of the potential categorical predictors (QST testing site, pain condition, pain type, type of pain induction stimulus) on the strength of the relationship between pain threshold and pain intensity/disability. The effect of each categorical predictor will be tested in a separate meta-regression.

The ability of QST to detect somatosensory differences between young people with chronic pain and healthy controls will be assessed using standardised mean differences (SMDs). The standardised mean difference will be calculated as the mean difference between groups divided by the standard deviation of the specific QST measure for all participants pooled across both groups. The standardised mean difference expresses the difference between groups in QST measures as multiples of the observed standard deviation. The standardised mean difference is a ratio, with numerator and

For peer review only - http://bmjopen.bmj.com/site/about/guidelines.xhtml

denominator in the same units as the original measurement, therefore has no units, does not depend on the original measurement scale, and allows a direct comparison of the effect across studies that used different QST measures. By convention, a SMD or $d = 0.2$ is considered to be small, $d= 0.5$ moderate and $d = 0.8$ large in size.[35] Each study will be screened for intraclass correlation coefficients (ICCs) describing the test-retest reliability of QST. The reliability of the measurement across test occasions will be rated excellent if the ICC > .75, adequate if .40 – .74, and poor if < .40.[36]

Patient and public involvement

Consultation with young people, parents and health care professionals has been used to determine their perception of the clinical utility of quantitative sensory testing in chronic pain. It is based on their perspectives that this systematic review was deemed timely and crucial to conduct to inform further research work and implementation of QST in routine clinical practice.

Discussion

This systematic review will be the first to investigate the association between QST and pain intensity or disability in paediatric chronic pain. The review findings will be used to inform our ongoing work to develop a core outcome set for specialist paediatric chronic pain services across the UK.

Ethics and dissemination

As this is a systematic review of published literature, ethical approval will not be sought. We will publish the protocol and our findings in peer-reviewed journals aimed at paediatric chronic pain clinicians and researchers as well as health commissioners. We will present our work at national and international meetings focused on paediatric chronic pain.

Acknowledgements

We would like to thank Holly Brown, Navin Down, Victoria Gibbons and Olivia Ranger (BSc Psychology students studying at the University of Southampton who undertook their 3rd year dissertation projects at the Pain Research Laboratory) for performing a scoping review of relevant literature that informed the development of this protocol.

For peer review only - http://bmjopen.bmj.com/site/about/guidelines.xhtml

Author Contributions

CL conceived the idea, planned and designed the study protocol. CL and DES planned the data extraction and statistical analysis and wrote the first draft; MB, JW, BZ, JZ and PB provided critical insights. All authors have approved and contributed to the final written manuscript.

Funding

This development of this protocol was supported by ESRC (Grant number: RES-000-22-4128). This funding source had no role in the design of this review and will not have any role during its execution, analyses, interpretation of the data, or decision to submit results.

Competing Interests

None declared.

For peer review only

References

1. Huguet A, Miró J. The severity of chronic pediatric pain: an epidemiological study. J Pain 2008;**9**(3):226-36.
2. Merskey H, & Bogduk, N. *Classification of chronic pain: Descriptions of chronic pain syndromes and definitions of pain terms*. Seattle: IASP Press 1994.
3. King S, Chambers CT, Huguet A, et al. The epidemiology of chronic pain in children and adolescents revisited: a systematic review. Pain 2011;**152**(12):2729-38 doi: 10.1016/j.pain.2011.07.016.
4. Tumin D, Drees D, Miller R, et al. Health Care Utilization and Costs Associated With Pediatric Chronic Pain. J Pain 2018;**19**(9):973-82 doi: 10.1016/j.jpain.2018.03.012.
5. Miró J, McGrath PJ, Finley GA, et al. Pediatric chronic pain programs: current and ideal practice. Pain Reports 2017;**2**(5):e613 doi: 10.1097/PR9.0000000000000613.
6. Rolfe PM. Paediatric chronic pain. Anaesthesia & Intensive Care Medicine 2016;**17**(11):531-35.
7. Varni JW, Limbers CA, Burwinkle TM. Impaired health-related quality of life in children and adolescents with chronic conditions: a comparative analysis of 10 disease clusters and 33 disease categories/severities utilizing the PedsQL 4.0 Generic Core Scales. Health Qual Life Outcomes 2007;**5**:43 doi: 10.1186/1477-7525-5-43.
8. Evans S, Djilas V, Seidman LC, et al. Sleep Quality, Affect, Pain, and Disability in Children With Chronic Pain: Is Affect a Mediator or Moderator? J Pain 2017;**18**(9):1087-95 doi: 10.1016/j.jpain.2017.04.007.
9. Liossi C, Howard RF. Pediatric chronic pain: biopsychosocial assessment and formulation. Pediatrics 2016;**138**(5):e20160331.
10. Rajapakse D, Liossi C, Howard RF. Presentation and management of chronic pain. Arch Dis Child 2014;**99**(5):474-80 doi: 10.1136/archdischild-2013-304207
11. Odell S, Logan DE. Pediatric pain management: the multidisciplinary approach. J Pain Res 2016;**6**:785–90. doi: 10.2147/JPR.S37434.

For peer review only - http://bmjopen.bmj.com/site/about/guidelines.xhtml

12. Liossi C, Johnstone L, Lilley S, et al. Interdisciplinary interventions for pediatric chronic pain: A systematic review and meta-analysis. Br J Anaesth in press.

13. Yarnitsky D. Quantitative Sensory Testing. Muscle Nerve 1997;**20**(2):198 - 204.

14. Yarnitsky D, Pud D. Quantitative sensory testing. In: Binnie CD, Cooper R, Mauguiere F, et al., eds. Clinical neurophysiology: EMG, nerve conduction and evoked potentials, vol1. Amsterdam: Elsevier, 2004:305 - 28.

15. Zaslansky R, Yarnitsky D. Clinical applications of quantitative sensory testing (QST). J Neurol Sci 1998;**153**(2):215-38 doi: https://doi.org/10.1016/S0022-510X(97)00293-1.

16. Hilz MJ, Glorius SE, Schweibold G, et al. Quantitative thermal perception testing in preschool children. Muscle & Nerve: Official Journal of the American Association of Electrodiagnostic Medicine 1996;**19**(3):381-83.

17. Tham SW, Palermo TM, Holley AL, et al. A population-based study of quantitative sensory testing in adolescents with and without chronic pain. Pain 2016;**157**(12):2807-15 doi: 10.1097/j.pain.0000000000000716.

18. Rolke R, Baron R, Maier C, et al. Quantitative sensory testing in the German Research Network on Neuropathic Pain (DFNS): Standardized protocol and reference values. Pain 2006;**123**(3):231-43.

19. Moher D, Shamseer L, Clarke M, et al. Preferred reporting items for systematic review and meta-analysis protocols (PRISMA-P) 2015 statement. Systematic Reviews 2015;**4**(1):1.

20. Shamseer L, Moher D, Clarke M, et al. Preferred reporting items for systematic review and meta-analysis protocols (PRISMA-P) 2015: elaboration and explanation. BMJ 2015;**349**:g7647 doi: 10.1136/bmj.g7647

21. Treede R-D, Rief W, Barke A, et al. A classification of chronic pain for ICD-11. Pain 2015;**156**(6):1003 doi: 0.1097/j.pain.0000000000000160.

22. Sawyer SM, Azzopardi PS, Wickremarathne D, et al. The age of adolescence. The Lancet: Child and Adolescent Health 2018;**2**(3):223 - 28 doi: 10.1016/S2352-4642(18)30022-1.

For peer review only - http://bmjopen.bmj.com/site/about/guidelines.xhtml

23. Liberati A, Altman DG, Tetzlaff J, et al. The PRISMA statement for reporting systematic reviews and meta-analyses of studies that evaluate health care interventions: explanation and elaboration. PLoS Med 2009;**6**(7):e1000100.

24. Downes MJ, Brennan ML, Williams HC, et al. Development of a critical appraisal tool to assess the quality of cross-sectional studies (AXIS). BMJ Open 2016;**6**(12):e011458 doi: 10.1136/bmjopen-2016-011458.

25. Von Elm E, Altman DG, Egger M, et al. The Strengthening the Reporting of Observational Studies in Epidemiology (STROBE) statement: guidelines for reporting observational studies. PLoS Med 2007;**4**(10):e296.

26. McHugh ML. Interrater reliability: The kappa statistic. Biochemia Medica 2012;**22**(3):276-82.

27. Higgins JP, Green S. *Cochrane handbook for systematic reviews of interventions*: John Wiley & Sons, 2011.

28. Blankenburg M, Boekens H, Hechler T, et al. Reference values for quantitative sensory testing in children and adolescents: Developmental and gender differences of somatosensory perception. Pain 2010;**149**(1):76-88 doi: http://dx.doi.org/10.1016/j.pain.2010.01.011.

29. Blankenburg M, Meyer D, Hirschfeld G, et al. Developmental and sex differences in somatosensory perception—a systematic comparison of 7- versus 14-year-olds using quantitative sensory testing. . Pain 2011;**152**(11):2625 - 31 doi: 10.1016/j.pain.2011.08.007.

30. Comprehensive Meta-Analysis Version 3 [program]. Englewood, New Jersey: Biostat, 2013.

31. Borenstein M, Hedges LV, Higgins J, et al. *Introduction to meta-analysis*. Cornwell: Wiley Online Library, 2009.

32. Hübscher M, Moloney N, Leaver A, et al. Relationship between quantitative sensory testing and pain or disability in people with spinal pain—a systematic review and meta-analysis. PAIN® 2013;**154**(9):1497-504.

33. Portney LG. *Foundations of Clinical Research: Applications to Practice 3th (third) Edition*. New Jersey: Pearson Education, 2009.

For peer review only - http://bmjopen.bmj.com/site/about/guidelines.xhtml

34. Higgins JPT, Green S. *Cochrane Handbook for Systematic Reviews of Interventions*: The Cochrane Collaboration, 2008.

35. Cohen J. *Statistical power analysis for the behavioral sciences, 2nd edition* Hillsdale: Erlbaum, 1988.

36. Fleiss JL, Levin B, Paik MC. *Statistical Methods for Rates and Proportions*: John Wiley & Sons, 2013.

For peer review only

PRISMA-P (Preferred Reporting Items for Systematic review and Meta-Analysis Protocols) 2015 checklist: recommended items to address in a systematic review protocol*

Section and topic	Item No	Checklist item	Page
**ADMINISTRATIVE INFORMATION**			
Title:			
Identification	1a	Identify the report as a protocol of a systematic review	1
Update	1b	If the protocol is for an update of a previous systematic review, identify as such	N/A
Registration	2	If registered, provide the name of the registry (such as PROSPERO) and registration number	6
Authors:			
Contact	3a	Provide name, institutional affiliation, e-mail address of all protocol authors; provide physical mailing address of corresponding author	1
Contributions	3b	Describe contributions of protocol authors and identify the guarantor of the review	11
Amendments	4	If the protocol represents an amendment of a previously completed or published protocol, identify as such and list changes; otherwise, state plan for documenting important protocol amendments	N/A
Support:			
Sources	5a	Indicate sources of financial or other support for the review	12
Sponsor	5b	Provide name for the review funder and/or sponsor	12
Role of sponsor or funder	5c	Describe roles of funder(s), sponsor(s), and/or institution(s), if any, in developing the protocol	12
**INTRODUCTION**			
Rationale	6	Describe the rationale for the review in the context of what is already known	4-5
Objectives	7	Provide an explicit statement of the question(s) the review will address with reference to participants, interventions, comparators, and outcomes (PICO)	5
**METHODS**			
Eligibility criteria	8	Specify the study characteristics (such as PICO, study design, setting, time frame) and report characteristics (such as years considered, language, publication status) to be used as criteria for eligibility for the review	6-8
Information sources	9	Describe all intended information sources (such as electronic databases, contact with study authors, trial registers or other grey literature sources) with planned dates of coverage	6
Search strategy	10	Present draft of search strategy to be used for at least one electronic database, including planned limits, such that it could be repeated	6

For peer review only

Study records:			
Data management	11a	Describe the mechanism(s) that will be used to manage records and data throughout the review	8-10
Selection process	11b	State the process that will be used for selecting studies (such as two independent reviewers) through each phase of the review (that is, screening, eligibility and inclusion in meta-analysis)	8
Data collection process	11c	Describe planned method of extracting data from reports (such as piloting forms, done independently, in duplicate), any processes for obtaining and confirming data from investigators	8
Data items	12	List and define all variables for which data will be sought (such as PICO items, funding sources), any pre-planned data assumptions and simplifications	6-8
Outcomes and prioritization	13	List and define all outcomes for which data will be sought, including prioritization of main and additional outcomes, with rationale	8-10
Risk of bias in individual studies	14	Describe anticipated methods for assessing risk of bias of individual studies, including whether this will be done at the outcome or study level, or both; state how this information will be used in data synthesis	8-10
Data synthesis	15a	Describe criteria under which study data will be quantitatively synthesised	8-10
	15b	If data are appropriate for quantitative synthesis, describe planned summary measures, methods of handling data and methods of combining data from studies, including any planned exploration of consistency (such as I^2, Kendall's τ)	8-11
	15c	Describe any proposed additional analyses (such as sensitivity or subgroup analyses, meta-regression)	10
	15d	If quantitative synthesis is not appropriate, describe the type of summary planned	N/A
Meta-bias(es)	16	Specify any planned assessment of meta-bias(es) (such as publication bias across studies, selective reporting within studies)	10
Confidence in cumulative evidence	17	Describe how the strength of the body of evidence will be assessed (such as GRADE)	8-9

*** It is strongly recommended that this checklist be read in conjunction with the PRISMA-P Explanation and Elaboration (cite when available) for important clarification on the items. Amendments to a review protocol should be tracked and dated. The copyright for PRISMA-P (including checklist) is held by the PRISMA-P Group and is distributed under a Creative Commons Attribution Licence 4.0.**

From: Shamseer L, Moher D, Clarke M, Ghersi D, Liberati A, Petticrew M, Shekelle P, Stewart L, PRISMA-P Group. Preferred reporting items for systematic review and meta-analysis protocols (PRISMA-P) 2015: elaboration and explanation. BMJ. 2015 Jan 2;349(jan02 1):g7647.

For peer review only
